# Reducing Aerosol-Related Risk of Transmission in the Era of COVID-19: An Interim Guidance Endorsed by the International Society of Aerosols in Medicine

**DOI:** 10.1089/jamp.2020.1615

**Published:** 2020-12-02

**Authors:** James B. Fink, Stephan Ehrmann, Jie Li, Patricia Dailey, Paul McKiernan, Chantal Darquenne, Andrew R. Martin, Barbara Rothen-Rutishauser, Philip J. Kuehl, Sabine Häussermann, Ronan MacLoughlin, Gerald C. Smaldone, Bernhard Muellinger, Timothy E. Corcoran, Rajiv Dhand

**Affiliations:** ^1^ Aerogen Pharma Corp., San Mateo, California, USA.; ^2^ Division of Respiratory Care, Department of Cardiopulmonary Sciences, Rush University Medical Center, Chicago, Illinois, USA.; ^3^ CHRU Tours, Médecine Intensive Réanimation, CIC INSERM 1415, CRICS-TriggerSep Research Network, Tours, France.; ^4^ INSERM, Centre d'étude des Pathologies Respiratoires, U1100, Université de Tours, Tours, France.; ^5^ Aerogen Limited, Galway, Ireland.; ^6^ Department of Medicine, University of California, San Diego, California, USA.; ^7^ Mechanical Engineering, University of Alberta, Edmonton, Canada.; ^8^ Adolphe Merkle Institute, University of Fribourg, Fribourg, Switzerland.; ^9^ Lovelace Biomedical, Albuquerque, New Mexico, USA.; ^10^ VisionHealth GmbH, Garching Forschungszentrum, Germany.; ^11^ School of Pharmacy and Biomolecular Sciences, Royal College of Surgeons, Dublin, Ireland.; ^12^ School of Pharmacy and Pharmaceutical Sciences, Trinity College, Dublin, Ireland.; ^13^ Pulmonary, Critical Care and Sleep Medicine, Department of Medicine, State University of New York at Stony Brook, Stony Brook, New York, USA.; ^14^ Vectura GmbH, Gauting, Germany.; ^15^ Division of Pulmonary, Allergy, and Critical Care Medicine, University of Pittsburgh, Pittsburgh, Pennsylvania, USA.; ^16^ Department of Medicine, Graduate School of Medicine, University of Tennessee Health Science Center, Knoxville, Tennessee, USA.

**Keywords:** aerosol generating procedures, bioaerosol dispersion, COVID-19, filters, medical aerosol, risk factors

## Abstract

National and international guidelines recommend droplet/airborne transmission and contact precautions for those caring for coronavirus disease 2019 (COVID-19) patients in ambulatory and acute care settings. The severe acute respiratory syndrome coronavirus 2 (SARS-CoV-2) virus, an acute respiratory infectious agent, is primarily transmitted between people through respiratory droplets and contact routes. A recognized key to transmission of COVID-19, and droplet infections generally, is the dispersion of bioaerosols from the patient. Increased risk of transmission has been associated with aerosol generating procedures that include endotracheal intubation, bronchoscopy, open suctioning, administration of nebulized treatment, manual ventilation before intubation, turning the patient to the prone position, disconnecting the patient from the ventilator, noninvasive positive-pressure ventilation, tracheostomy, and cardiopulmonary resuscitation. The knowledge that COVID-19 subjects can be asymptomatic and still shed virus, producing infectious droplets during breathing, suggests that health care workers (HCWs) should assume every patient is potentially infectious during this pandemic. Taking actions to reduce risk of transmission to HCWs is, therefore, a vital consideration for safe delivery of all medical aerosols. Guidelines for use of personal protective equipment (glove, gowns, masks, shield, and/or powered air purifying respiratory) during high-risk procedures are essential and should be considered for use with lower risk procedures such as administration of uncontaminated medical aerosols. Bioaerosols generated by infected patients are a major source of transmission for SARS CoV-2, and other infectious agents. In contrast, therapeutic aerosols do not add to the risk of disease transmission unless contaminated by patients or HCWs.

Coronavirus disease 2019 (COVID-19) is a viral pandemic affecting >200 countries. Severe acute respiratory syndrome coronavirus 2 (SARS-CoV-2), the virus that causes COVID-19, belongs to the same class of coronaviruses as those that resulted in SARS and Middle East respiratory syndrome (MERS), both of which infected many health care workers (HCWs) in the course of providing patient care.^(1)^ The new SARS-CoV-2 virus, an acute respiratory infectious agent, is primarily transmitted between people through respiratory droplets and contact routes.^(2,3)^ A recognized key to transmission of COVID-19, and droplet infections generally, is the dispersion of bioaerosols from the patient. Droplets generated by infected persons when they cough, sneeze, talk, sing, or breathe range from 0.1 to >100 μm in diameter.^(4–6)^ They can land in the mouth, nose, or eyes of those in proximity, and they have the potential to be inhaled into the lungs. However, larger droplets settle on surfaces around the infected subject, where they could be infectious by contact for several days.^(7)^ It is not clear that contact with surfaces is a major way for transmission for COVID-19.^(8,9)^ Smaller droplets when airborne may form droplet nuclei that can be carried farther away from the source and are highly respirable; although airborne transmission of COVID-19 between individuals over long distances has been shown, this does not appear to be the major route of transmission of infection.^(6,10)^

Droplet transmission is by no means unique to COVID-19. Acute respiratory infections, particularly of the lower respiratory tract, are the leading cause of morbidity and mortality from infectious disease globally, accounting for >4 million deaths annually.^(10)^ Although bacteria are a common cause of lower respiratory tract infections, the majority are caused by viruses or a mix of viral/bacterial infections, all of which can be exhaled by infected patients as bioaerosols.

National and international guidelines recommend droplet/airborne and contact precautions for those caring for COVID-19 patients in ambulatory and acute care settings.^(1,11)^ Increased risk of transmission has been associated with aerosol generating procedures (AGPs) that include endotracheal intubation, bronchoscopy, open suctioning, administration of nebulized treatment, manual ventilation before intubation, turning the patient to the prone position, disconnecting the patient from the ventilator, noninvasive positive-pressure ventilation, tracheostomy, and cardiopulmonary resuscitation. Some of these procedures increase the production of bioaerosols possibly containing pathogens from the patient (i.e., intubation, open suctioning, tracheotomy, manual ventilation, and bronchoscopy), whereas others potentially act to disperse bioaerosols from the patient to the surrounding area without evidence that they generate additional contaminated aerosols (e.g., oxygen administration, high-flow nasal oxygen, and use of medical nebulizers). In response to the surge of the current pandemic, and given the lack of expertise of many HCWs concerning medical aerosols and their interactions with bioaerosol production, a group of International Society for Aerosols in Medicine (ISAM) members presented an initial draft to the board, and a panel including some of the board members was established to draft a short interim guidance document to address the most important points and concerns, with the intent to convene a wider team to provide a more detailed analysis of background and issues related to AGPs at a later date. This guidance is not discouraging the use of pressurized metered dose inhaler (pMDI) or inhalers that are appropriate in many circumstances, but provides rationale for appropriate use of nebulizers that are equally appropriate in many circumstances.

Medical aerosols produced by inhalers and nebulizers, such as those containing bronchodilators, anti-inflammatory agents, mucokinetics, antivirals, antibiotics, and prostanoids, do not contain pathogens unless the nebulizers are contaminated by the patient or HCW. Medical aerosols from nebulization derive from a nonpatient source (the fluid in the nebulizer chamber) and have not been shown to carry patient-derived viral particles. Concerns of medical aerosol becoming contaminated in the lungs before exhalation are not supported by evidence. Consequently, when a droplet in the aerosol coalesces with a contaminated mucous membrane, it will cease to be airborne and, therefore, will no longer be part of an aerosol.^(12)^ In fact, aerosol administration has been reported to reduce generation of bioaerosols. Edwards and colleagues reported that inhalation of an isotonic saline aerosol reduced generation of bioaerosols by as much as 72% for up to 6 hours postnebulization presumably associated with change in fluid characteristics in the airway.^(13)^

Many AGPs were identified during previous outbreaks of SARS, MERS, and other viral infections such as influenza A.^(14,15)^ Early response of the SARS outbreak in Hong Kong was to ban all medical aerosols, which they categorized as AGPs.^(14)^ Retrospective analysis of SARS reports and research identified pooled analysis of risk for a variety of AGPs, with intubation and noninvasive manual ventilation creating a 6.6- and 3.3-fold increased risk of infection of HCWs, respectively.^(16)^ In contrast, the pooled risk from medical nebulizer treatment from three cohort reports was considered nonsignificant (0.9).^(16)^ Nevertheless, HCWs should learn how to reduce risks associated with all aerosol delivery devices as not all drugs that are nebulized in the acute care setting are available in pMDIs, dry powder inhalers (DPIs), or soft mist inhalers (SMIs) such as antibiotics and prostaglandins.^(17,18)^

Inhalers, such as pMDIs, have been suggested to present less risk than other medical aerosols, often without supporting evidence.^(19,20)^ The rationale for reduced risk compared with other medical aerosols may be because drug is enclosed and less open to contamination than open cup nebulizers, and the low emitted dose (100 μL/actuation) produces less aerosol mass with shorter treatment times.^(21,22)^ As with electronic/mechanical nebulizers/inhalers that only emit aerosol during inspiration, exhaled bioaerosols whether from cough or normal exhalation are neither avoided nor contained with use of pMDIs, SMIs, or DPIs. Characteristics of drug formulation can precipitate cough with both nebulizers and inhalers.^(23)^ The mechanism of bioaerosol generation during cough associated with inhalers or medical aerosols from nebulizers is similar to cough, that is, independent of inhaled medication and likely generates as much bioaerosol. Consequently, inhalers offer no innate advantage in reducing production or dispersion of patient-generated bioaerosols. Even with use of valved holding chambers, patient exhalation exhausts directly to the atmosphere unless there is a mechanism to filter the exhaled breath. This is not to say that inhalers should not be used, but the ability of inhalers to reduce bioaerosol transmission has not been established.

Medical nebulizer treatments may increase the mass and dispersion of aerosol^(24–26)^; however, there is no evidence that medical nebulizers increase the infective load of bioaerosols unless the nebulizer is contaminated. Historically, concerns of nebulizer contamination were focused on jet nebulizers with open reservoirs positioned below the ventilator circuit or mouthpiece, which present a risk of contamination by secretions, condensate, and even bioaerosols.^(27)^ Once medication in the nebulizer is contaminated the resulting aerosol emitted may increase the viral load with adverse consequences for both the patient and the environment. This is the basis of recommendations by The Centers for Disease Control and Prevention (CDC) that jet nebulizers be replaced, rinsed, air dried, washed, disinfected, and/or sterilized after each treatment.^(28)^ In addition, continuous jet nebulizers driven with flows up to 10 L/min may increase the dispersion of aerosol.^(29)^ Breath-synchronized jet nebulizers, which produce aerosol only during inspiration, reduce fugitive emissions compared with nebulizers that operate continuously during the breathing cycle.^(30)^ During mechanical ventilation opening or “breaking” the pressurized circuit to add medication or clean nebulizers is associated with explosive depressurization known to generate possibly infectious aerosols from condensate in the circuit, in addition to interrupting life support. This can be minimized by the use of valved T adapter that allows a nebulizer to be removed without depressurizing the ventilator circuit.

By design, vibrating mesh nebulizers (VMNs) separate the medication from the patient interface, including breathing circuits by the barrier of the mesh. This mesh maintains pressure in the ventilator circuit when the medication reservoir is opened to add medication, without a measurable leak of gas through the nebulizer to atmosphere, allowing medication to be added without “breaking” the ventilator circuit. In addition, the medication reservoir of the VMN is positioned above the circuit, reducing the potential for gravity-dependent contamination from condensate in the circuit and patient-generated secretions.^(31)^ As VMNs do not use external gas flow to generate aerosol, they are less likely to contribute to dispersion of patient-generated bioaerosol beyond that from the patient's exhalation.^(32)^

Independent of the type of nebulizer used, a risk of bioaerosol dispersion exists in case of contamination of the reservoir during the medication loading process, which needs to be performed using aseptic techniques. In contrast, inhalers with contained doses do not carry this risk. In all instances, one should limit administration of aerosol formulations and solutions that precipitate cough, including distilled water and hypertonic saline.

Independent of aerosol device selection, placing filters on nebulizers and expiratory ports of devices reduces fugitive emissions of infected bioaerosols generated by the patient and they reduce secondhand exposure of HCWs to aerosol medication.^(33)^ Similarly, placing a filter on the expiratory limb of a ventilator circuit reduces escape of bioaerosols and medical aerosols, thereby reducing the risk of transmitting infection. Proprietary filters placed distal to the expiratory port of ventilators reduced fugitive emissions to 0.25% of aerosol generated, whereas placement of a simple filter in the expiratory limb allowed 40%–55% of fugitive aerosol to exit the ventilator.^(34)^

Owing to the required gas flow, dispersion of bioaerosols with oxygen administration, including venturi, simple, and nonrebreather masks all have the potential to disperse bioaerosols from the patient farther than a medical jet nebulizer.^(28)^ Placing a filter on the outlet of a nebulizer greatly reduces dispersion of all aerosols.^(33,34)^ Placing a surgical mask over a nasal cannula, whether low or high flow, reduces dispersion of patient-generated bioaerosols as well as medical aerosols.^(35,36)^ Having patients wear surgical masks can reduce the risk of transmission to HCWs.^(37,38)^

The knowledge that COVID-19 subjects can be asymptomatic and still shed virus,^(39)^ producing infectious droplets during breathing, suggests that HCWs should assume every patient is potentially infectious during this pandemic.^(40)^ Taking actions to reduce risk of transmission to HCWs is, therefore, a vital consideration for safe delivery of all medical aerosols. Guidelines for use of personal protective equipment (PPE) (gloves, gowns, masks, shield, and/or powered air purifying respirators [PAPRs]) during high-risk procedures are essential and should be considered for use with lower risk procedures such as administration of uncontaminated medical aerosols.^(1,11,40)^

Recommendations:

Bioaerosols generated by infected patients are a major potential source of transmission for SARS CoV-2, and other infectious agents.^(40)^ The risk of disease transmission by therapeutic medical aerosol could be mitigated by taking certain precautions as mentioned hereunder:

- During the pandemic treat every patient as potentially infected because asymptomatic infected patients can shed virus.^(41,42)^
- Use PPE for aerosol and droplet protection (mask, face shield, gloves, and gown).^(40)^
- Wash hands and put on fresh gloves before filling the nebulizer reservoir and administering treatments. Use proper aseptic technique to avoid contamination of aerosol reservoirs and medication.
- Perform high-risk AGPs in a negative pressure room, if available, for COVID-19 patients, or rooms with high air exchange rates (6–12/hour), and use additional PPE such as PAPRs.^(1,11,40)^
- Have patients wear simple mask when possible [i.e., over simple nasal cannula and high flow nasal cannula (HFNC)] and between treatments.
- Have tissues available and encourage covering cough or sneeze with tissues; discard used tissue immediately,
- Reduce dispersion of aerosols.

Use mouthpiece with handheld applications when possible, since both open and valved aerosol masks release more aerosol to atmosphere and are harder to filter.^(33)^

Social distancing: Maintaining distance of 1 m or more from patient reduces risk of transmission.^(40)^ when treating patients try to stay >45 cm (maximum dispersion distance with oxygen and medical aerosol) away from the patient's airway.^(35)^

Minimize release of medical aerosols, to minimize secondhand exposure of medication and dispersion of bioaerosols into the environment; use a valved chamber in which medical aerosol is contained until inhalation, or a breath synchronized nebulizer that does not generate aerosol after inhalation.^(29)^

Place filter on exhalation port of nebulizers, single limb noninvasive ventilator circuits, and dual-limb critical care ventilators.^(32,33)^

Avoid “breaking” open the ventilator circuit to add medication or change nebulizers, as this generates aerosol from condensate that may be infectious^(28)^; if needed with jet nebulizer, add a valved T-adapter in-line with ventilator circuit.

Aerosol can be administered through HFNC; with lower dispersion than open aerosol masks.^(38)^

A surgical mask placed over oxygen cannulas, nose and mouth acts as a barrier to contain bioaerosols generated and reduce dispersion distance.^(35,36)^

Medical nebulizers should be disposed of, rinsed, air dried, washed, or sterilized between treatments or if VMN cleaned based on manufacturer label.^(28,32)^

Note: HCWs should comply with the requirements and guidelines of their region and institution.

## Authors' Contributions

J.B.F., S.E., and J.L. coauthored the first draft guidance document. P.D. and P.M.K. provided background on guidances and references. All authors provided input, revised multiple drafts, and approved the document.

## Notes

Reviewed by:

Anonymous
